# Supplementary material for: Multi-tissue expression and splicing data prioritise anatomical subsite- and sex-specific colorectal cancer susceptibility genes
Source: Nat Commun. 2025 May 30;16:5043. doi: 10.1038/s41467-025-60275-6 (PMC12125321; doi:10.1038/s41467-025-60275-6)
Supplement: Supplementary file 1 — Supplementary Information [file 41467_2025_60275_MOESM1_ESM.pdf]

Multi-tissue expression and splicing data prioritise anatomical subsite- and sex-specific colorectal cancer susceptibility genes

Supplementary Information

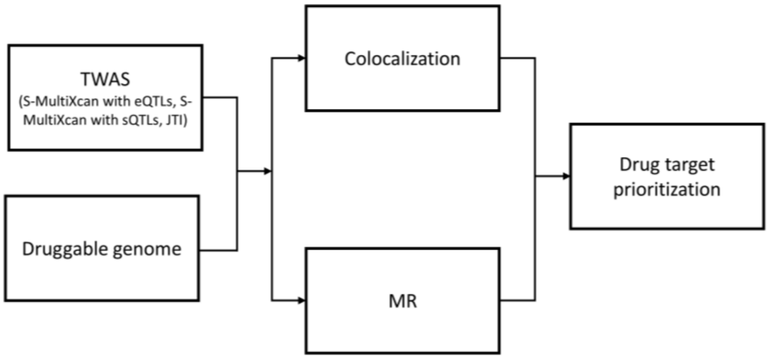

Supplementary Fig 1. Flowchart detailing analysis plan. JTI = joint tissue imputation; eQTLs = expression quantitative trait loci; sQTLs = splice quantitative trait loci; MR = Mendelian randomization.

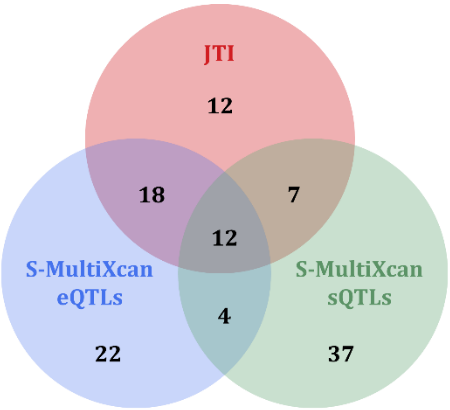

Supplementary Fig 2. Venn diagram showing overlap of genes identified by each TWAS analysis. JTI = joint tissue imputation; eQTLs = expression quantitative trait loci; sQTLs = splice quantitative trait loci.

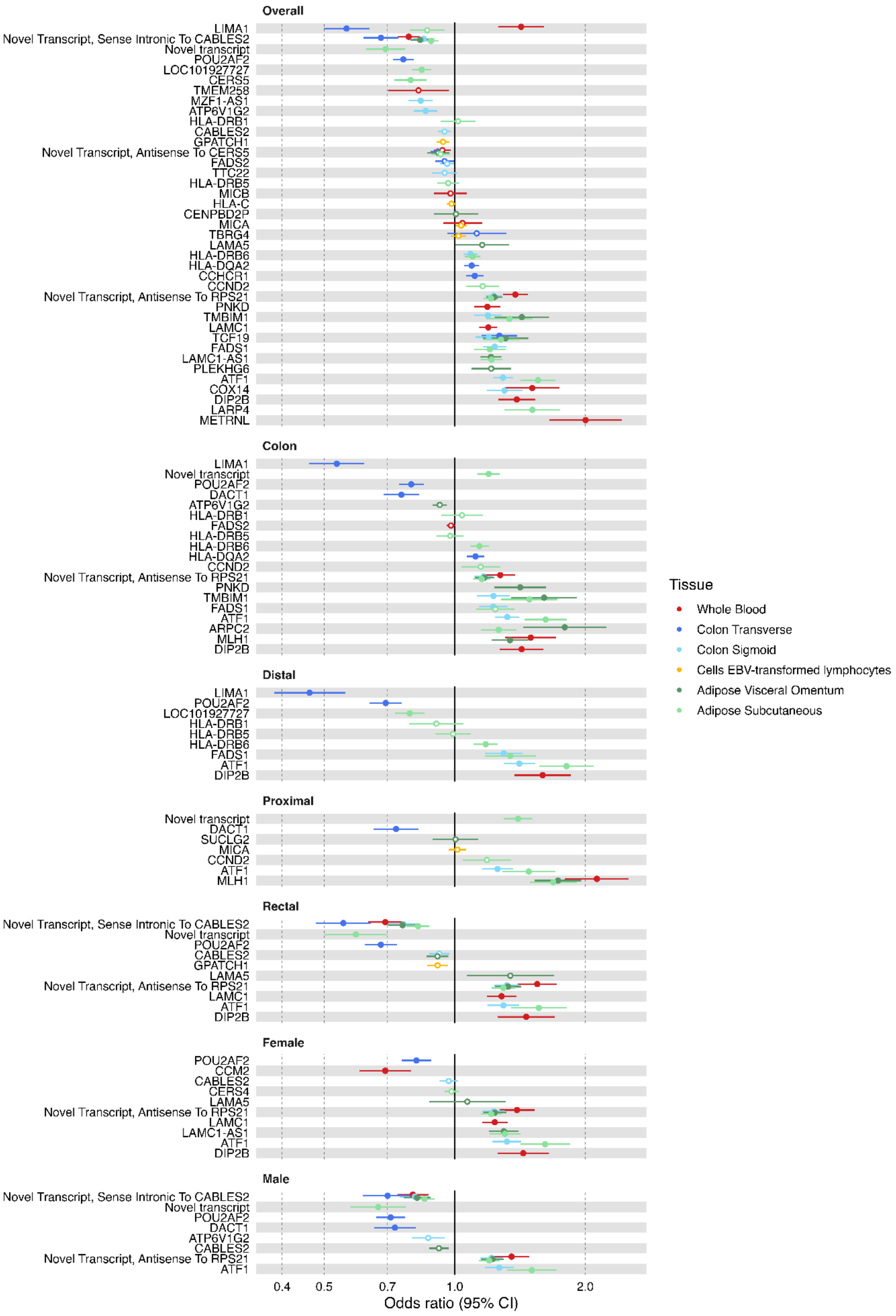

Overall
LIMA1
Novel Transcript, Sense Intronic To CABLES2
Novel transcript
POU2AF2
LOC101927727
CERS5
TMEM258
MZF1-AS1
ATP6V1G2
HLA-DRB1
CABLES2
GPATCH1
Novel Transcript, Antisense To CERS5
FADS2
TTC22
HLA-DRB5
MICB
HLA-C
CENPBD2P
MICA
TBRG4
LAMA5
HLA-DRB6
HLA-DQA2
CCHCR1
CCND2
Novel Transcript, Antisense To RPS21
PNKD
TMBIM1
LAMC1
TCF19
FADS1
LAMC1-AS1
PLEKHG6
ATF1
COX14
DIP2B
LARP4
METRNL
Colon
LIMA1
Novel transcript
POU2AF2
DACT1
ATP6V1G2
HLA-DRB1
FADS2
HLA-DRB5
HLA-DRB6
HLA-DQA2
CCND2
Novel Transcript, Antisense To RPS21
PNKD
TMBIM1
FADS1
ATF1
ARPC2
MLH1
DIP2B
Distal
LIMA1
POU2AF2
LOC101927727
HLA-DRB1
HLA-DRB5
HLA-DRB6
FADS1
ATF1
DIP2B
Proximal
Novel transcript
DACT1
SUCLG2
MICA
CCND2
ATF1
MLH1
Rectal
Novel Transcript, Sense Intronic To CABLES2
Novel transcript
POU2AF2
CABLES2
GPATCH1
LAMA5
Novel Transcript, Antisense To RPS21
LAMC1
ATF1
DIP2B
Female
POU2AF2
CCM2
CABLES2
CERS4
LAMA5
Novel Transcript, Antisense To RPS21
LAMC1
LAMC1-AS1
ATF1
DIP2B
Male
Novel Transcript, Sense Intronic To CABLES2
Novel transcript
POU2AF2
DACT1
ATP6V1G2
CABLES2
Novel Transcript, Antisense To RPS21
ATF1
0.4
0.5
0.7
1.0
2.0
Odds ratio (95% CI)
Tissue
Whole Blood
Colon Transverse
Colon Sigmoid
Cells EBV-transformed lymphocytes
Adipose Visceral Omentum
Adipose Subcutaneous

Supplementary Fig 3. Forest plot showing results from MR analyses of expression of genes from seven tissues identified by TWAS analysis on colorectal cancer risk. Solid points indicate the Bonferroni p-value threshold of $P < 4.38 \times 10^{-5}$ (0.05/N*G where N is the number of gene-tissue pairs (161), and G is the number of CRC GWAS (7)) was met in the MR analysis. CI = confidence interval.

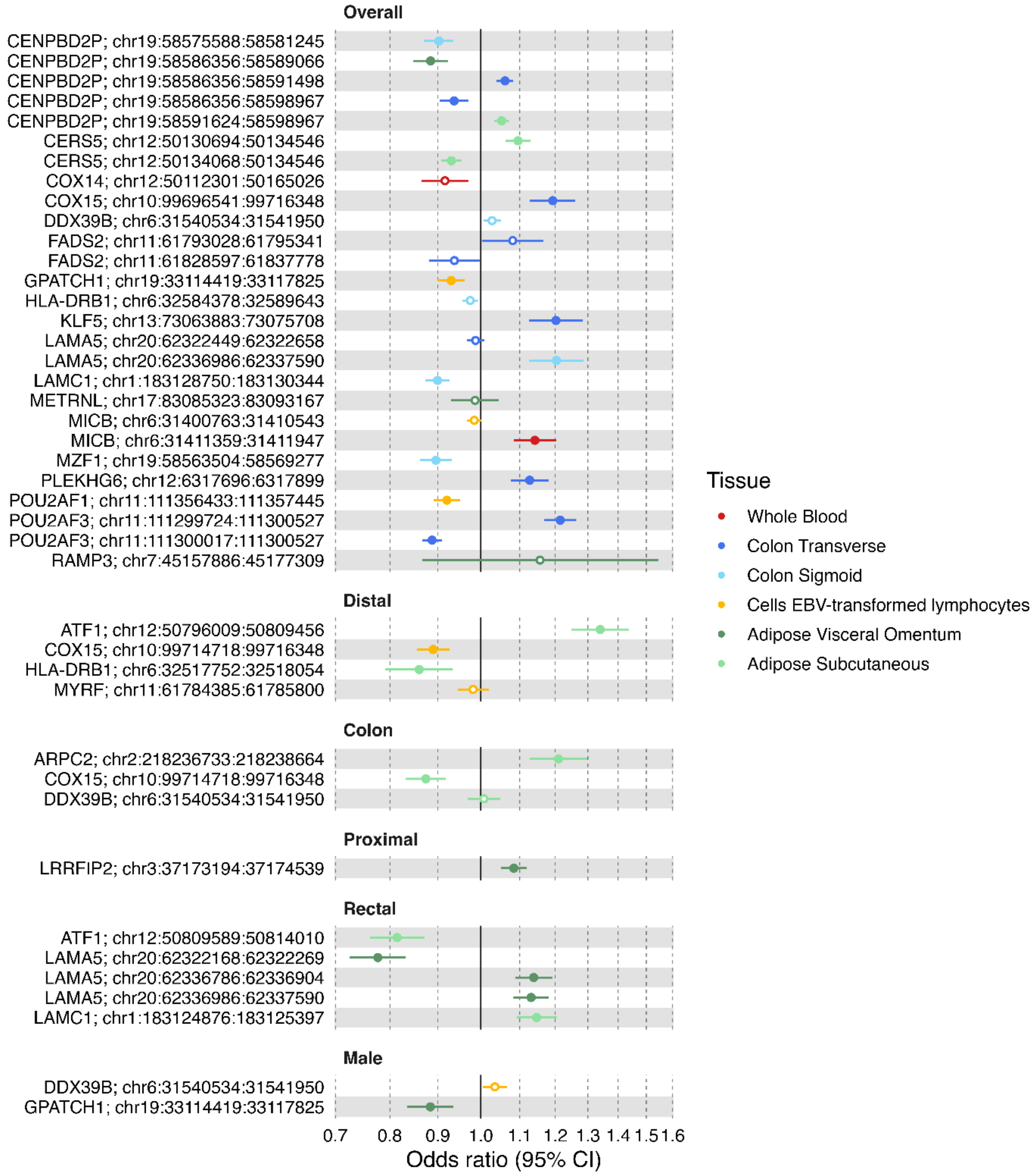

Supplementary Fig 4. Forest plot showing results from MR analyses of splicing events from seven tissues identified by TWAS analysis on colorectal cancer risk. Solid points

indicate the Bonferroni p-value threshold of $P < 1.19 \times 10^{-3}$ (0.05/ 42, the number of unique splicing event-tissue-subtype trios with suitable instruments for MR analyses) was met in the MR analysis. CI = confidence interval.

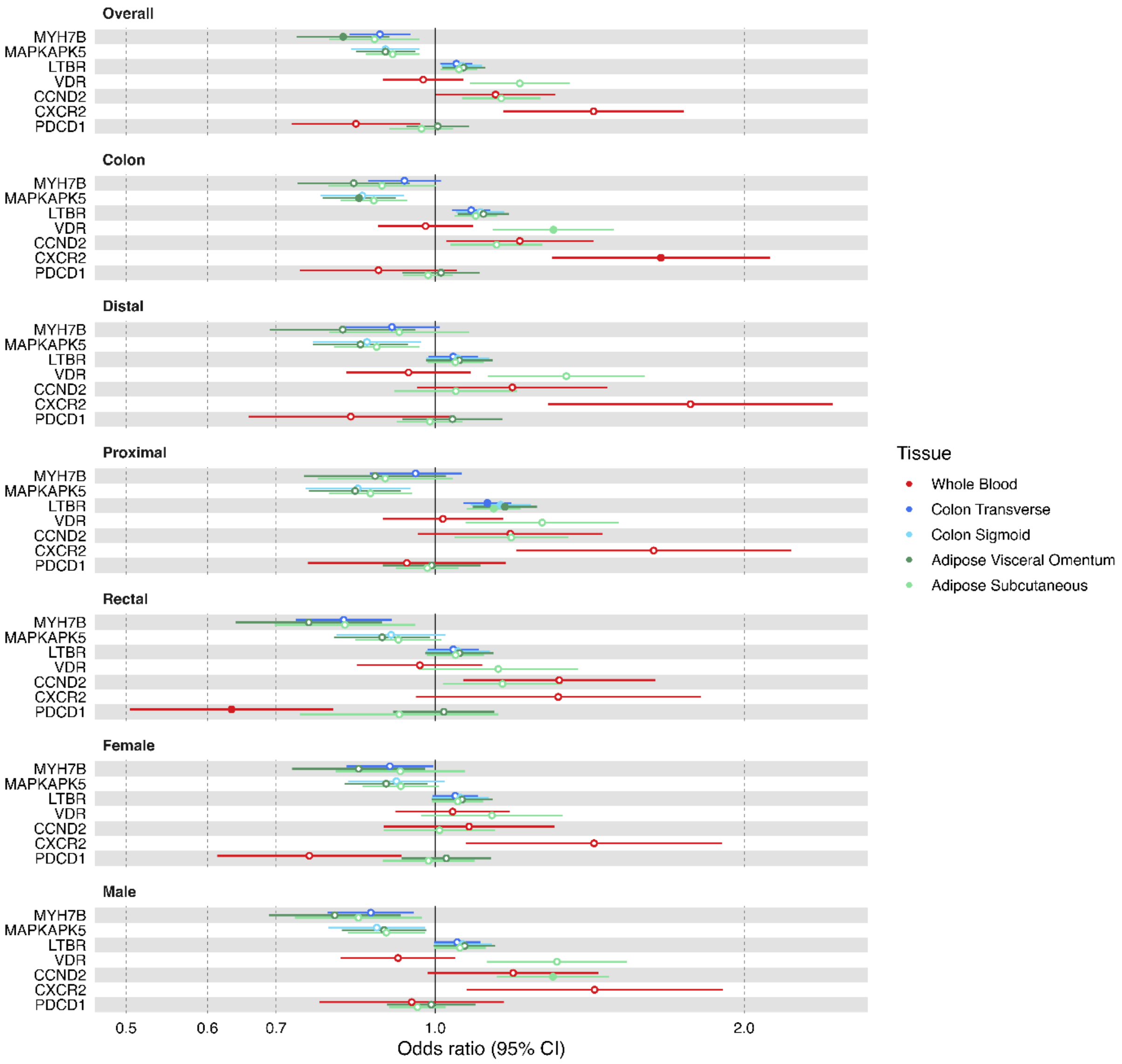

Supplementary Fig 5. Forest plot showing results from MR analyses of the "druggable genome" on colorectal cancer risk. Only genes which met the Bonferroni *P*-value threshold of $P < 1.32 \times 10^{-4}$ (0.05/number of druggable genes with suitable genetic instruments available (380)) in any MR analysis are shown. Solid points indicate this Bonferroni p-value threshold was met in the MR analysis. CI = confidence interval.

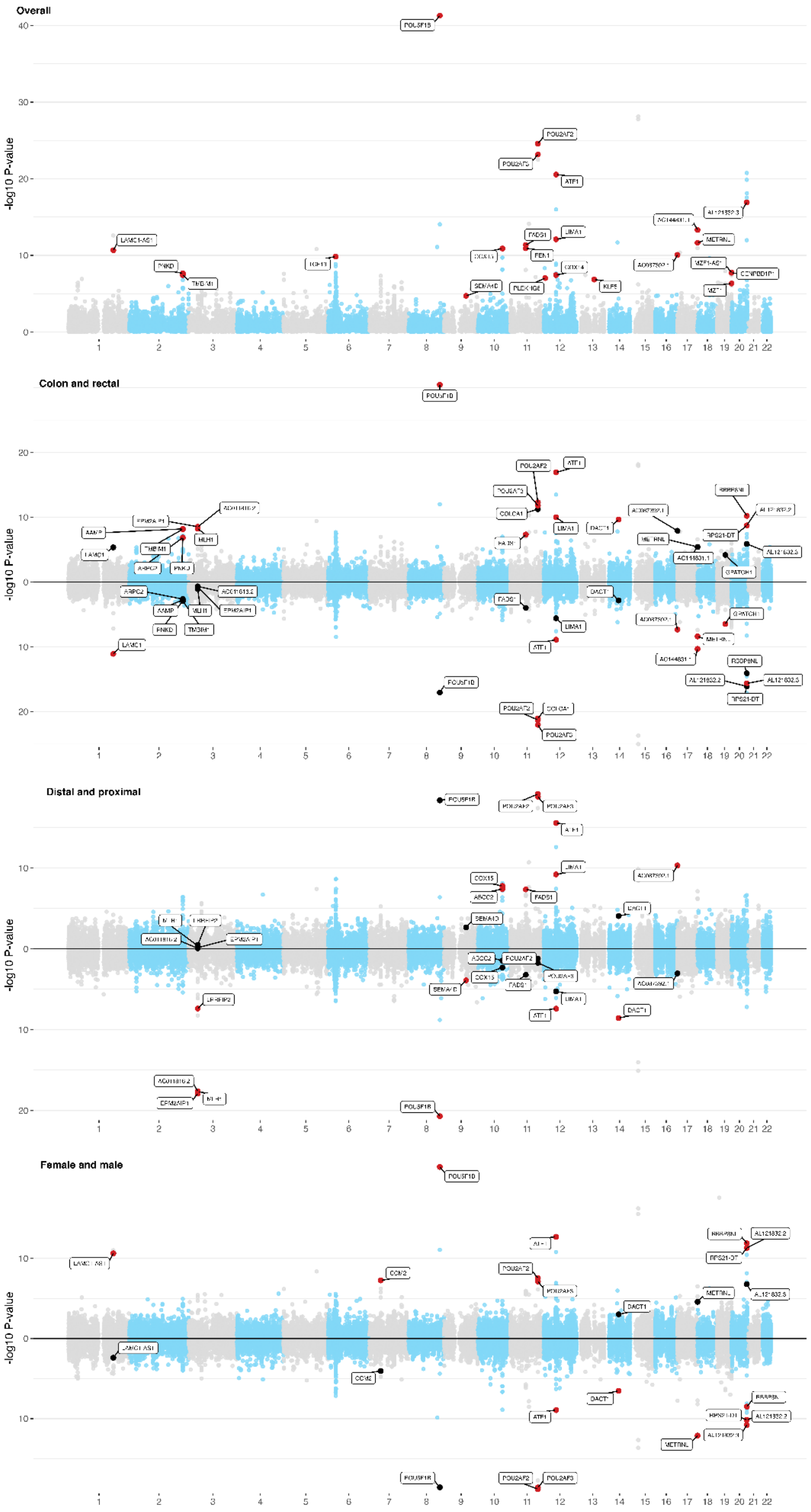
Overall
Colon and rectal
Distal and proximal
Female and male
-log10 P-value
POU5F1B
POU2AF2
POU2AF3
ATF1
LIMA1
FADS1
DACT1
METRNL
CENPBD1P1
RPS21-DT
GPATCH1
SEMA4D
COX15
ABCC2
EPHA2AP1
CCM2

Supplementary Fig 6. Manhattan and Miami plots showing results of S-MultiXcan and JTI TWAS analyses for each anatomical subsite of colorectal cancer. Where genes were identified in multiple TWAS analyses, the one with the lowest *P*-value was retained. Genes labelled are those prioritised following subsequent analyses. Red = prioritised genes for this anatomical subsite; black = prioritised genes for the other anatomical subsite in the Miami plot.

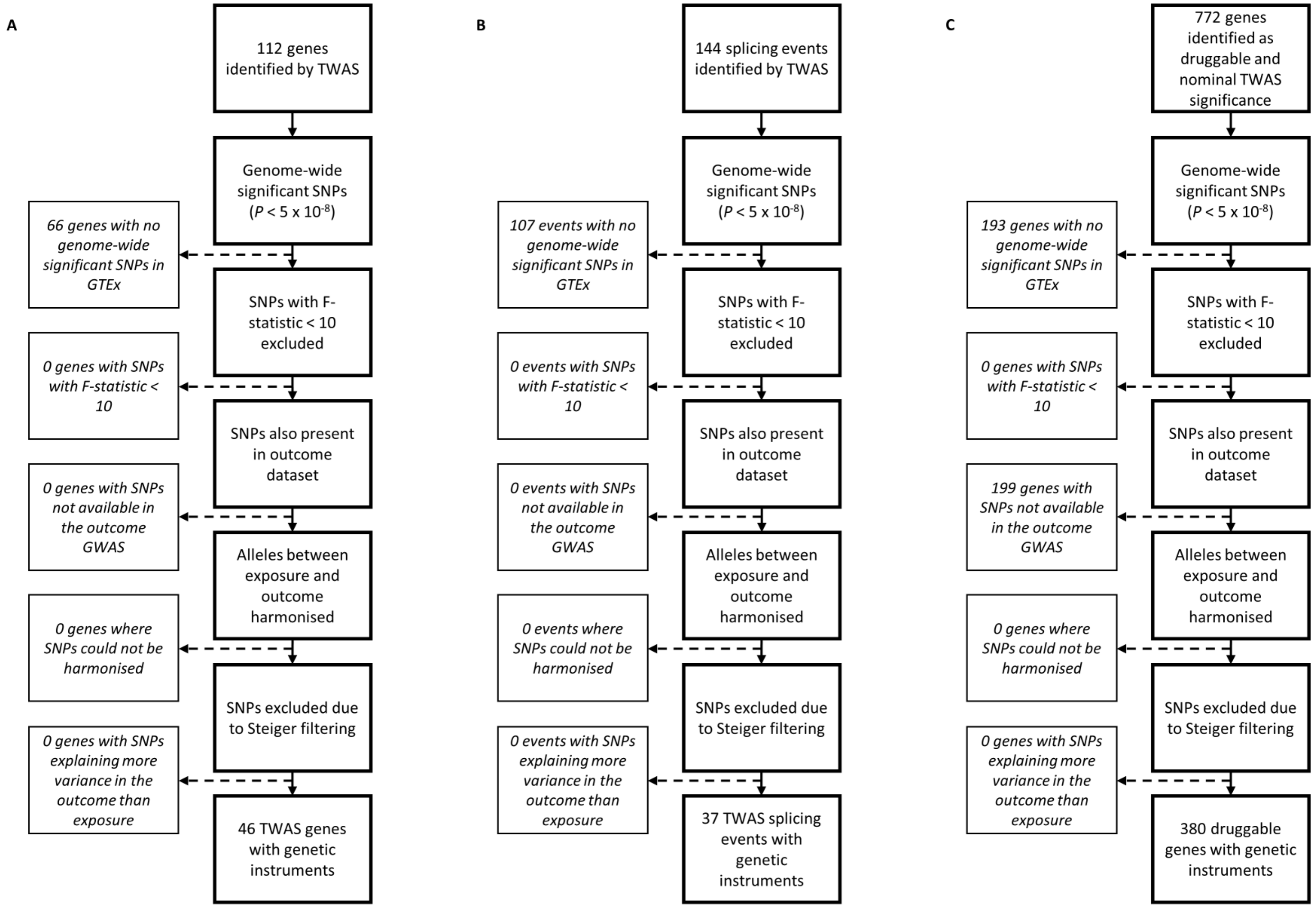
A
112 genes identified by TWAS
Genome-wide significant SNPs ($P < 5 \times 10^{-8}$)
66 genes with no genome-wide significant SNPs in GTEx
SNPs with F-statistic < 10 excluded
0 genes with SNPs with F-statistic < 10
SNPs also present in outcome dataset
0 genes with SNPs not available in the outcome GWAS
Alleles between exposure and outcome harmonised
0 genes where SNPs could not be harmonised
SNPs excluded due to Steiger filtering
0 genes with SNPs explaining more variance in the outcome than exposure
46 TWAS genes with genetic instruments
B
144 splicing events identified by TWAS
Genome-wide significant SNPs ($P < 5 \times 10^{-8}$)
107 events with no genome-wide significant SNPs in GTEx
SNPs with F-statistic < 10 excluded
0 events with SNPs with F-statistic < 10
SNPs also present in outcome dataset
0 events with SNPs not available in the outcome GWAS
Alleles between exposure and outcome harmonised
0 events where SNPs could not be harmonised
SNPs excluded due to Steiger filtering
0 events with SNPs explaining more variance in the outcome than exposure
37 TWAS splicing events with genetic instruments
C
772 genes identified as druggable and nominal TWAS significance
Genome-wide significant SNPs ($P < 5 \times 10^{-8}$)
193 genes with no genome-wide significant SNPs in GTEx
SNPs with F-statistic < 10 excluded
0 genes with SNPs with F-statistic < 10
SNPs also present in outcome dataset
199 genes with SNPs not available in the outcome GWAS
Alleles between exposure and outcome harmonised
0 genes where SNPs could not be harmonised
SNPs excluded due to Steiger filtering
0 genes with SNPs explaining more variance in the outcome than exposure
380 druggable genes with genetic instruments

Supplementary Fig 7. Flowchart showing genetic instrument construction and the number of traits (i.e. gene expression or slice events) that were excluded at each stage. A. Construction of genetic instruments to proxy gene expression for genes identified by TWAS. **B.** Construction of genetic instruments to proxy splicing events. **C**. Construction of genetic instruments to proxy gene expression of the druggable genome. TWAS = transcriptome-wide association study; SNPs = single nucleotide polymorphisms.

STROBE-MR checklist of recommended items to address in reports of Mendelian randomization studies[1 2]

Item No.	Section	Checklist item	Page No.	Relevant text from manuscript
1	**TITLE and ABSTRACT**	Indicate Mendelian randomization (MR) as the study's design in the title and/or the abstract if that is a main purpose of the study	4	involving Mendelian randomisation
	**INTRODUCTION**			
2	**Background**	Explain the scientific background and rationale for the reported study. What is the exposure? Is a potential causal relationship between exposure and outcome plausible? Justify why MR is a helpful method to address the study question	5	no CRC TWAS performed thus far has stratified by anatomical subsite or sex, which are important aspects of CRC development. Additionally, TWAS for CRC have often lacked a causal framework analysis
3	**Objectives**	State specific objectives clearly, including pre-specified causal hypotheses (if any). State that MR is a method that, under specific assumptions, intends to estimate causal effects	6, 12	genes, we performed comprehensive multi-tissue TWAS analyses as outlined in **Figure 1**. We then employed a causal framework performing Mendelian randomization (MR)<br>Under certain assumptions, MR can provide causal estimates (see **Methods**)
	**METHODS**			
4	**Study design and data sources**	Present key elements of the study design early in the article. Consider including a table listing sources of data for all phases of the study. For each data source contributing to the analysis, describe the following:		

		a)	Setting: Describe the study design and the underlying population, if possible. Describe the setting, locations, and relevant dates, including periods of recruitment, exposure, follow-up, and data collection, when available.	14	Summary genetic association data for CRC risk (52,775 cases, 45,940 controls)
		b)	Participants: Give the eligibility criteria, and the sources and methods of selection of participants. Report the sample size, and whether any power or sample size calculations were carried out prior to the main analysis	14	(52,775 cases, 45,940 controls)
		c)	Describe measurement, quality control and selection of genetic variants	16	We identified genetic instruments as genetic variants which are *cis*-acting (i.e. within 100kb of the gene coding region), strongly associated with gene expression ($P < 5 \times 10^{-8}$), independent ($r^2 < 0.001$), and had an F-statistic > 10
		d)	For each exposure, outcome, and other relevant variables, describe methods of assessment and diagnostic criteria for diseases	7	Briefly, proximal, distal and rectal are mutually exclusive anatomical subsites designated by location of tumour, whereas colon is comprised of proximal colon and distal colon tumours, as well as colon cancer with unspecified location.
		e)	Provide details of ethics committee approval and participant informed consent, if relevant		
5	**Assumptions**		Explicitly state the three core IV assumptions for the main analysis (relevance, independence and exclusion restriction) as well assumptions for any additional or sensitivity analysis	15	The three core assumptions of MR state that: (i) the genetic variant(s) are strongly and robustly associated with the exposure; (ii) there is no confounding of the genetic variant(s)-outcome relationship (e.g., population stratification); (iii) the genetic

				variant(s) only affect the outcome through their effect on the exposure.
6	**Statistical methods: main analysis**	Describe statistical methods and statistics used		
	a)	Describe how quantitative variables were handled in the analyses (i.e., scale, units, model)	18	Units of gene expression betas, as outlined by the GTEx consortium, are the result of a normalisation procedure consisting of normalisation between samples using the trimmed mean of M values (TMM) method, followed by normalisation across samples by inverse normal transformation, and as such the normalised expression units have no direct biological interpretation (see https://gtexportal.org/home/methods for more information)
	b)	Describe how genetic variants were handled in the analyses and, if applicable, how their weights were selected		
	c)	Describe the MR estimator (e.g. two-stage least squares, Wald ratio) and related statistics. Detail the included covariates and, in case of two-sample MR, whether the same covariate set was used for adjustment in the two samples	16	Where only a single genetic variant was available, we calculated the Wald ratio to generate effect estimates; where multiple genetic variants were available, an inverse variable weighted (IVW) multiplicative random effects model was used.
	d)	Explain how missing data were addressed		

		e)	If applicable, indicate how multiple testing was addressed	16	A Bonferroni-correction was applied to account for multiple testing ($P < 4.38 \times 10^{-5}$; 0.05/N*G where N is the number of gene-tissue pairs (161) and G is the number of CRC GWAS (overall, male, female, colon, distal, proximal, rectal).
7	**Assessment of assumptions**		Describe any methods or prior knowledge used to assess the assumptions or justify their validity		
8	**Sensitivity analyses and additional analyses**		Describe any sensitivity analyses or additional analyses performed (e.g. comparison of effect estimates from different approaches, independent replication, bias analytic techniques, validation of instruments, simulations)		
9	**Software and pre-registration**				
		a)	Name statistical software and package(s), including version and settings used	18	"Statistical analyses" section
		b)	State whether the study protocol and details were pre-registered (as well as when and where)		
	**RESULTS**				
10	**Descriptive data**				
		a)	Report the numbers of individuals at each stage of included studies and reasons for exclusion. Consider use of a flow diagram		
		b)	Report summary statistics for phenotypic exposure(s), outcome(s), and other relevant variables (e.g. means, SDs, proportions)		
		c)	If the data sources include meta-analyses of previous studies, provide the assessments of heterogeneity across these studies		
		d)	For two-sample MR:	14	All almost entirely European ancestry, no individuals likely to overlap.

			i. Provide justification of the similarity of the genetic variant-exposure associations between the exposure and outcome samples<br>ii. Provide information on the number of individuals who overlap between the exposure and outcome studies		
11	**Main results**				
		a)	Report the associations between genetic variant and exposure, and between genetic variant and outcome, preferably on an interpretable scale		Supplementary table 13
		b)	Report MR estimates of the relationship between exposure and outcome, and the measures of uncertainty from the MR analysis, on an interpretable scale, such as odds ratio or relative risk per SD difference		Supplementary tables 4 and 5
		c)	If relevant, consider translating estimates of relative risk into absolute risk for a meaningful time period		
		d)	Consider plots to visualize results (e.g. forest plot, scatterplot of associations between genetic variants and outcome versus between genetic variants and exposure)		Figures 3 and 4
12	**Assessment of assumptions**				
		a)	Report the assessment of the validity of the assumptions	7, 12	Genetic colocalisation analysis assesses whether the same or distinct variant(s) underly the association between two traits<br>many of our MR analyses were restricted to a single SNP, meaning we were unable to employ various “pleiotropy-robust” models to evaluate exclusion restriction assumptions
		b)	Report any additional statistics (e.g., assessments of heterogeneity across genetic variants, such as I^2, Q statistic or E-value)		

13	**Sensitivity analyses and additional analyses**				
		a)	Report any sensitivity analyses to assess the robustness of the main results to violations of the assumptions		
		b)	Report results from other sensitivity analyses or additional analyses		Results of TWAS and colocalization throughout results section
		c)	Report any assessment of direction of causal relationship (e.g., bidirectional MR)		
		d)	When relevant, report and compare with estimates from non-MR analyses		
		e)	Consider additional plots to visualize results (e.g., leave-one-out analyses)		
	**DISCUSSION**				
14	**Key results**		Summarize key results with reference to study objectives	10	Using this framework, we identified 35 genes with a likely causal association
15	**Limitations**		Discuss limitations of the study, taking into account the validity of the IV assumptions, other sources of potential bias, and imprecision. Discuss both direction and magnitude of any potential bias and any efforts to address them	12	Our analysis aimed to robustly prioritise CRC susceptibility genes specific to sex and anatomical subsite by using multiple tissues alongside a causal framework. However…
16	**Interpretation**				
		a)	Meaning: Give a cautious overall interpretation of results in the context of their limitations and in comparison with other studies	12	Our analyses have identified genes with robust evidence for a potential causal role in CRC development, providing insights into its aetiology and presenting tangible opportunities for targeted therapeutic interventions.

		b) Mechanism: Discuss underlying biological mechanisms that could drive a potential causal relationship between the investigated exposure and the outcome, and whether the gene-environment equivalence assumption is reasonable. Use causal language carefully, clarifying that IV estimates may provide causal effects only under certain assumptions	Throughout discussion	e.g. Cerebral cavernous malformation 2 (CCM2) is a component of the CCM signalling complex, which has a role in regulating several signalling cascades, including progesterone signalling
		c) Clinical relevance: Discuss whether the results have clinical or public policy relevance, and to what extent they inform effect sizes of possible interventions	12	Given the increase in CRC worldwide, understanding the biological mechanisms leading to carcinogenesis is becoming increasingly important. Additionally, as more screening programmes are rolled out globally, opportunities to prevent CRC development in high-risk individuals are also increasing. Therefore, the identification of novel pharmaceutical targets for the prevention and treatment of this disease remains a priority
17	**Generalizability**	Discuss the generalizability of the study results (a) to other populations, (b) across other exposure periods/timings, and (c) across other levels of exposure	12	our analysed were restricted to individuals of predominantly European ancestries, which limits the generalisability of our findings to other populations and contexts
	**OTHER INFORMATION**			
18	**Funding**	Describe sources of funding and the role of funders in the present study and, if applicable, sources of funding for the databases and original study or studies on which the present study is based	19	Acknowledgements section

19	**Data and data sharing**	Provide the data used to perform all analyses or report where and how the data can be accessed, and reference these sources in the article. Provide the statistical code needed to reproduce the results in the article, or report whether the code is publicly accessible and if so, where	18	All data generated by this analysis can be found within the manuscript and supporting information, or the online repository on Zenodo
20	**Conflicts of Interest**	All authors should declare all potential conflicts of interest	20	Declaration of interests section

This checklist is copyrighted by the Equator Network under the Creative Commons Attribution 3.0 Unported (CC BY 3.0) license.

1. Skrivankova VW, Richmond RC, Woolf BAR, Yarmolinsky J, Davies NM, Swanson SA, et al. Strengthening the Reporting of Observational Studies in Epidemiology using Mendelian Randomization (STROBE-MR) Statement. JAMA. 2021;under review.

2. Skrivankova VW, Richmond RC, Woolf BAR, Davies NM, Swanson SA, VanderWeele TJ, et al. Strengthening the Reporting of Observational Studies in Epidemiology using Mendelian Randomisation (STROBE-MR): Explanation and Elaboration. BMJ. 2021;375:n2233.
