# Supplementary material for: Multi-tissue expression and splicing data prioritise anatomical subsite- and sex-specific colorectal cancer susceptibility genes
Source: Nat Commun. 2025 May 30;16:5043. doi: 10.1038/s41467-025-60275-6 (PMC12125321; doi:10.1038/s41467-025-60275-6)
Supplement: Supplementary file 2 — Description of Additional Supplementary Files [file 41467_2025_60275_MOESM2_ESM.pdf]

Description of Additional Supplementary Files

Supplementary Data 1. Results from S-MultiXcan with splicing quantitative trait loci (sQTLs).

Supplementary Data 2. Results from S-MultiXcan with expression quantitative trait loci (eQTLs).

Supplementary Data 3. Results from JTI with expression quantitative trait loci (eQTLs).

Supplementary Data 4. Results from MR of TWAS-identified genes and colorectal cancer risk.

Supplementary Data 5. Results from MR of TWAS-identified splicing events and colorectal cancer risk.

Supplementary Data 6. Results from MR of the “druggable genome” and colorectal cancer risk.

Supplementary Data 7. Results of colocalisation of TWAS-identified genes with expression quantitative trait loci (eQTLs) and colorectal cancer risk.

Supplementary Data 8. Results of colocalisation of TWAS-identified genes with splicing quantitative trait loci (sQTLs) and colorectal cancer risk.

Supplementary Data 9. Results of colocalisation of “druggable genome” with expression quantitative trait loci (eQTLs) and colorectal cancer risk.

Supplementary Data 10. Results of colocalisation using Pairwise Conditional Colocalisation (PWCoCo) of TWAS-identified and druggable genes with expression quantitative trait loci (eQTLs) and colorectal cancer risk.

Supplementary Data 11. Results of colocalisation using Pairwise Conditional Colocalisation (PWCoCo) of TWAS-identified genes with splicing quantitative trait loci (sQTLs) and colorectal cancer risk.

Supplementary Data 12. Results of the functional enrichment analysis of the 37 likely causal susceptibility genes using g:Profiler.

Supplementary Data 13. Results from splicing event annotation analysis using SpliceAI.

Supplementary Data 14. Results from the BioGRID Open Repository on CRISPR Screens analysis.

Supplementary Data 15. Results of colocalisation of expression of identified genes and risk factors.

Supplementary Data 16. Details of the GWAS used in all analyses.

Supplementary Data 17. Genetic instruments used in all MR analyses.
