# Supplementary material for: Multi-tissue expression and splicing data prioritise anatomical subsite- and sex-specific colorectal cancer susceptibility genes
Source: Nat Commun. 2025 May 30;16:5043. doi: 10.1038/s41467-025-60275-6 (PMC12125321; doi:10.1038/s41467-025-60275-6)
Supplement: Supplementary file 4 — Reporting Summary [file 41467_2025_60275_MOESM4_ESM.pdf]

nature portfolio

Corresponding author(s): Tracy A O'Mara

Last updated by author(s): Apr 1, 2025

# Reporting Summary

Nature Portfolio wishes to improve the reproducibility of the work that we publish. This form provides structure for consistency and transparency in reporting. For further information on Nature Portfolio policies, see our Editorial Policies and the Editorial Policy Checklist.

## Statistics

For all statistical analyses, confirm that the following items are present in the figure legend, table legend, main text, or Methods section.

| n/a | Confirmed | |
|---|---|---|
| ☐ | ☒ | The exact sample size ($n$) for each experimental group/condition, given as a discrete number and unit of measurement |
| ☐ | ☒ | A statement on whether measurements were taken from distinct samples or whether the same sample was measured repeatedly |
| ☐ | ☒ | The statistical test(s) used AND whether they are one- or two-sided<br>*Only common tests should be described solely by name; describe more complex techniques in the Methods section.* |
| ☐ | ☒ | A description of all covariates tested |
| ☐ | ☒ | A description of any assumptions or corrections, such as tests of normality and adjustment for multiple comparisons |
| ☐ | ☒ | A full description of the statistical parameters including central tendency (e.g. means) or other basic estimates (e.g. regression coefficient) AND variation (e.g. standard deviation) or associated estimates of uncertainty (e.g. confidence intervals) |
| ☐ | ☒ | For null hypothesis testing, the test statistic (e.g. $F$, $t$, $r$) with confidence intervals, effect sizes, degrees of freedom and $P$ value noted<br>*Give P values as exact values whenever suitable.* |
| ☐ | ☒ | For Bayesian analysis, information on the choice of priors and Markov chain Monte Carlo settings |
| ☒ | ☐ | For hierarchical and complex designs, identification of the appropriate level for tests and full reporting of outcomes |
| ☐ | ☒ | Estimates of effect sizes (e.g. Cohen's $d$, Pearson's $r$), indicating how they were calculated |

*Our web collection on statistics for biologists contains articles on many of the points above.*

## Software and code

Policy information about availability of computer code

| | |
|---|---|
| Data collection | No software was used for data collection. |
| Data analysis | Units of gene expression betas, as outlined by the GTEx consortium, are the result of a normalisation procedure consisting of normalisation between samples using the trimmed mean of M values method, followed by normalisation across samples by inverse normal transformation, and as such the normalised expression units have no direct biological interpretation (see https://gtexportal.org/home/methods for more information). All analyses were performed using R version 4.0.2 or Python version 3.9.13 (other than the GWAS imputation step of the TWAS analysis which was performed using version 3.5.0). The following R packages were used: for colocalisation analyses, coloc (version 5.1.0.1); for MR analyses TwoSampleMR (version 0.5.5), gwasglue (version 0.0.0.9000); for compiling LD reference panels, plinkbinr (version 0.0.0.9000 ), ieugwasr (version 0.1.5); for accessing Ensembl databases, biomaRt (version 2.46.3); for finemapping, susieR (version 0.12.35). All code is publicly available on GitHub at: https://github.com/EmmaHazelwood/CRC-TWAS-code and archived on Zenodo: (DOI: 10.5281/zenodo.12805738). |

For manuscripts utilizing custom algorithms or software that are central to the research but not yet described in published literature, software must be made available to editors and reviewers. We strongly encourage code deposition in a community repository (e.g. GitHub). See the Nature Portfolio guidelines for submitting code & software for further information.

## Data

Policy information about availability of data

All manuscripts must include a data availability statement. This statement should provide the following information, where applicable:

- Accession codes, unique identifiers, or web links for publicly available datasets
- A description of any restrictions on data availability
- For clinical datasets or third party data, please ensure that the statement adheres to our policy

The data generated in this study can be found within the manuscript and supporting information, or the online respository on the Zenodo database under accession code 12805739 (https://doi.org/10.5281/zenodo.12805739). The CRC GWAS data used in this analysis are from GECCO. Individual-level data, CCFR, CORECT, CORSA_2 and GECCO are deposited in dbGaP (accession numbers phs001415.v1.p1, phs001315.v1.p1, phs001078.v1.p1, phs001903.v1.p1, phs001856.v1.p1 and phs001045.v1.p1). CCFR 1 and CCFR 2 data can be requested by submitting an application for collaboration to the CCFR (forms, instructions and contact information can be located at www.coloncfr/collaboration.org). For access to CORSA_1, please contact gecco@fredhutch.org. For more information see: https://research.fredhutch.org/peters/en/genetics-and-epidemiology-of-colorectal-cancer-consortium.html. The precomputed PrediXcan models were downloaded from http://predictdb.org) and pretrained JTI models were downloaded from Zenodo (https://doi.org/10.5281/zenodo.3842289). Source data for all figures in the manuscript are provided as Supplementary Data.

## Research involving human participants, their data, or biological material

Policy information about studies with human participants or human data. See also policy information about sex, gender (identity/presentation), and sexual orientation and race, ethnicity and racism.

| | |
|---|---|
| Reporting on sex and gender | Analyses stratified by sex was performed using provided colorectal cancer GWAS summary statistics. Sex was defined based on sex chromosomes and samples with with discrepancies between reported and genotypic sex based on X chromosome heterozygosity were excluded. See Huyghe, J. R. et al. Discovery of common and rare genetic risk variants for colorectal cancer. Nat Genet 51, 76–87 2019 & Huyghe, J. R. et al. Genetic architectures of proximal and distal colorectal cancer are partly distinct. Gut 70, 1325–1334 2021 for further details. |
| Reporting on race, ethnicity, or other socially relevant groupings | All participants in the anatomical subsite-specific colorectal analyses were of genetically inferred European ancestries. Approximately 92% of participants in the overall colorectal GWAS were European and ~8% were from East Asian populations. GWAS summary statistics were not provided stratified by these variables. |
| Population characteristics | Summary genetic association data for CRC risk (52,775 cases, 45,940 controls) were obtained from a meta-analysis of the Colorectal Transdisciplinary Study (CORECT), the Colon Cancer Family Registry (CCFR), and the Genetics and Epidemiology of CRC (GECCO) consortium. Supplementary Table 16 shows all GWAS studies used in these analyses. References for further details are provided. |
| Recruitment | This study used summary-level and not individual-level data. All study participants were recruited by previous studies. |
| Ethics oversight | The colorectal GWAS from which the summary statistics were derived had obtained informed consent from all participants and had obtained approval from their research ethics committees or institutional review boards (see Huyghe, J. R. et al. Discovery of common and rare genetic risk variants for colorectal cancer. Nat Genet 51, 76–87 2019 & Huyghe, J. R. et al. Genetic architectures of proximal and distal colorectal cancer are partly distinct. Gut 70, 1325–1334 2021). |

Note that full information on the approval of the study protocol must also be provided in the manuscript.

# Field-specific reporting

Please select the one below that is the best fit for your research. If you are not sure, read the appropriate sections before making your selection.

[x] Life sciences  [ ] Behavioural & social sciences  [ ] Ecological, evolutionary & environmental sciences

For a reference copy of the document with all sections, see nature.com/documents/nr-reporting-summary-flat.pdf

# Life sciences study design

All studies must disclose on these points even when the disclosure is negative.

| | |
|---|---|
| Sample size | No sample calculations were required in advance. Sample sizes were determined by accessing summary statistics from one of the largest GWAS for colorectal cancer, and precomputed models from large eQTL and sQTL datasets. This ensured most statistically powerful analyses possible were performed. |
| Data exclusions | No data were excluded. |
| Replication | No replication phase was included in this study. Our study is based on one of the largest GWAS for colorectal cancer and there is not another independent replication set without overlapping samples that is available. However, stringent correction for multiple testing was applied throughout the analysis pipeline to minimise type I error. |
| Randomization | NA. Randomisation is not applicable given we have used summary statistics for GWAS and prediction models. |

| Blinding | NA. Blinding is not applicable given we have used summary statistics for GWAS and prediction models. |
|---|---|

# Reporting for specific materials, systems and methods

We require information from authors about some types of materials, experimental systems and methods used in many studies. Here, indicate whether each material, system or method listed is relevant to your study. If you are not sure if a list item applies to your research, read the appropriate section before selecting a response.

## Materials & experimental systems

| n/a | Involved in the study |
|---|---|
| ☒ | ☐ Antibodies |
| ☒ | ☐ Eukaryotic cell lines |
| ☒ | ☐ Palaeontology and archaeology |
| ☒ | ☐ Animals and other organisms |
| ☒ | ☐ Clinical data |
| ☒ | ☐ Dual use research of concern |
| ☒ | ☐ Plants |

## Methods

| n/a | Involved in the study |
|---|---|
| ☒ | ☐ ChIP-seq |
| ☒ | ☐ Flow cytometry |
| ☒ | ☐ MRI-based neuroimaging |

# Plants

| | |
|---|---|
| Seed stocks | *Report on the source of all seed stocks or other plant material used. If applicable, state the seed stock centre and catalogue number. If plant specimens were collected from the field, describe the collection location, date and sampling procedures.* |
| Novel plant genotypes | *Describe the methods by which all novel plant genotypes were produced. This includes those generated by transgenic approaches, gene editing, chemical/radiation-based mutagenesis and hybridization. For transgenic lines, describe the transformation method, the number of independent lines analyzed and the generation upon which experiments were performed. For gene-edited lines, describe the editor used, the endogenous sequence targeted for editing, the targeting guide RNA sequence (if applicable) and how the editor was applied.* |
| Authentication | *Describe any authentication procedures for each seed stock used or novel genotype generated. Describe any experiments used to assess the effect of a mutation and, where applicable, how potential secondary effects (e.g. second site T-DNA insertions, mosiacism, off-target gene editing) were examined.* |
